# Antineuroinflammatory and Neuroprotective Effects of Gyejibokryeong-Hwan in Lipopolysaccharide-Stimulated BV2 Microglia

**DOI:** 10.1155/2019/7585896

**Published:** 2019-04-01

**Authors:** Bo-Kyung Park, Young Hwa Kim, Yu Ri Kim, Jeong June Choi, Changsop Yang, Ik-Soon Jang, Mi Young Lee

**Affiliations:** ^1^Clinical Medicine Division, Korea Institute of Oriental Medicine, Daejeon 34054, Republic of Korea; ^2^Laboratory of Molecular Medicine, College of Korean Medicine, Daejeon University, Daejeon 34520, Republic of Korea; ^3^Division of Bioconvergence Analysis, Korea Basic Science Institute, Daejeon 34133, Republic of Korea

## Abstract

Microglia, the central nervous system's innate immune cells, mediate neuroinflammation and are implicated in a variety of neuropathologies. The present study investigated the antineuroinflammatory and neuroprotective effects of Gyejibokryeong-hwan (GBH), a traditional Korean medicine, in lipopolysaccharide- (LPS-) stimulated murine BV2 microglia. BV2 cells were pretreated with GBH, fluoxetine (FXT), or amitriptyline (AMT) for 1 h and then stimulated with LPS (100 ng/mL). The expression levels of nitric oxide (NO), cytokines, and chemokines were determined by the Griess method, ELISA, or real-time PCR. Western blotting was used to measure various transcription factors and mitogen activated protein kinase (MAPK) and phosphatidylinositol 3-kinase (PI3K)/Akt activity. GBH significantly reduced the levels of NO, inducible nitric oxide synthase (iNOS), cyclooxygenase- (COX-) 2, tumor necrosis factor- (TNF-) *α*, interleukin- (IL-) 1*β*, IL-6, macrophage inhibitory protein- (MIP-) 1*α*, macrophage chemoattractant protein- (MCP-) 1, and IFN-*γ* inducible protein- (IP-) 10, regulated upon activation normal T cell expressed sequence (RANTES) in a dose-dependent manner. Expression of nuclear factor- (NF-) *κ*B p65 was significantly decreased and phosphorylation of extracellular signal-regulated kinase (Erk), c-Jun NH2-terminal kinase (JNK), and PI3K/Akt by GBH, but not p38 MAPK, was decreased. Furthermore, production of anti-inflammatory cytokine IL-10 was increased and Heme oxygenase-1 (HO-1) was upregulated via the nuclear factor-E2-related factor 2 (NRF2)/cAMP response element-binding protein (CREB) pathway, collectively indicating the neuroprotective effects of GBH. We concluded that GBH may suppress neuroinflammatory responses by inhibiting NF-*κ*B activation and upregulating the neuroprotective factor, HO-1. These results suggest that GBH has potential as anti-inflammatory and neuroprotective agents against microglia-mediated neuroinflammatory disorders.

## 1. Introduction

Microglia are the resident immune cells of the central nervous system (CNS) and act as an early defense system against proinflammatory reactions in the brain and prevent some effects of aging and repair damaged tissues [1, 2]. However, when microglia are activated by sustained stimulation, they can secrete a variety of inflammatory factors including proinflammatory cytokines such as tumor necrosis factor- (TNF-) *α*, interleukin- (IL-) 1*β* and IL-6, chemokines including macrophage inhibitory protein- (MIP-) 1*α*, monocyte chemoattractant protein 1- (MCP-) 1, the interferon- (IFN-) *γ* inducible protein- (IP-) 10, regulated upon activation normal T cell expressed sequence (RANTES), and reactive oxidants [2, 3]. When these inflammatory factors are chronically secreted, they can cause neuroinflammation, as in various neurodegenerative disorders such as Alzheimer's disease, Parkinson's disease, depression, and amyotrophic lateral sclerosis (ALS) [4].

Lipopolysaccharide- (LPS-) stimulated microglia activate Toll-like receptor (TLR), phosphorylate mitogen-activated protein kinase (MAPK), and translocate nuclear factor- (NF-) *κ*B p65, an inflammatory transcription factor, into the nucleus via phosphatidylinositol 3-kinase (PI3K)/Akt, where it increases the production of various proinflammatory cytokines and chemokines [5, 6]. In contrast, an increase in neuroprotective factors such as nuclear factor erythroid 2-related factor 2 (NRF2), cAMP response element-binding protein (CREB), and Heme oxygenase-1 (HO-1) can produce the anti-inflammatory cytokine IL-10 and thus inhibit neuroinflammation. Given this central role of microglia in neuroinflammation, controlling the activation of microglia cells through upregulation of HO-1 may serve as a critical potential target mechanism for the treatment of neurological and neurodegenerative diseases [7, 8].

Gyejibokryeong-hwan (GBH) is a traditional Korean medicine that is described in Donguibogam, a textbook of traditional Korean medicines [9]. GBH has been extensively used throughout Asia in the treatment of blood stasis [10, 11] and is approved by the Ministry of Food and Drug safety (MFDS) and the USA Food and Drug Administration (FDA), having been studied for safety and composition. GBH contains herbs including* Cinnamomum cassia *Presl,* Poria cocos *Wolf,* Paeonia suffruticosa *Andrews,* Paeonia lactiflora *Pallas, and* Prunus persica *Batsch [12, 13].

*Cinnamomum cassia *Presl, a constitutive agent in GBH, has been shown to exhibit anti-inflammatory effects in murine BV2 microglia cells, and* Paeonia suffruticosa *Andrews, another element of GBH, has been reported to induce antioxidative stress effects in rat pheochromocytoma PC-12 cells [14, 15]. Moreover, amygdalin, a component of* Prunus persica *Batsch, induces neurotrophic effects via the activation of the extracellular signal-regulated kinase (ERK)1/2 pathway in PC12 cells [16], paeoniflorin, a component of* Paeonia suffruticosa *Andrews and* Paeonia lactiflora *Pallas, reduces proinflammatory cytokines in CNS via the activation of Akt-NF-*κ*B signaling pathway* in vivo *and* in vitro* [17], and cinnamic acid, a component of* Cinnamomum cassia *Presl, upregulates the expression of SOCS3 in glial cells via the CREB pathway [18].

In a previous study conducted by our group, GBH was shown to exhibit antineuroinflammation effects in the hippocampus of a mouse model of reserpine-induced depression [19]. Several studies have reported that microglia regulate hippocampal neurogenesis in neurodegeneration [20, 21]. Despite these promising results, the antineuroinflammation and neuroprotection effects of GBH in murine BV2 microglia have yet to be investigated. Therefore, in the present study, we confirmed the antineuroinflammatory and neuroprotective effects of GBH via several mechanisms in a proinflammatory microglia model where murine BV2 microglia were treated with LPS.

## 2. Materials and Methods

### 2.1. Preparation of Gyejibokryeong-Hwan (GBH)

GBH was purchased from Hanpoong Pharm and Foods Co., Ltd. (Jeonju, Korea). A voucher specimen of the herb sample was deposited in the Herbarium of Hanpoong Pharm and Foods Co., Ltd. (voucher no. 17248). The five-component herbs of GBH (combined at a 1:1:1:1:1 ratio; total weight = 1.7 kg) were boiled in water for 3 h to create an extract which was then filtered and concentrated via vacuum pressure. The extract yield was approximately 29.53%. The extract was stored at -80°C and dissolved in phosphate-buffered saline (PBS) before use.

### 2.2. Reagents and Liquid Chromatography/Mass Spectrometry (LC/MS) Analysis of Gyejibokryeong-Hwan

#### 2.2.1. Amygdalin and Paeoniflorin

Standards for amygdalin (PubChem CID: 656516, purity 99.0%) and paeoniflorin (PubChem CID: 442534, purity 98.8%) were purchased from Sigma-Aldrich (St. Louis, MO, USA). Solutions of amygdalin and paeoniflorin were prepared in analytical grade methanol (Merck, Darmstadt, Germany). An optimized multiple reaction monitoring (MRM) method was developed using Ultra-Performance Liquid Chromatography (UPLC) coupled with tandem mass spectrometry (MS/MS). A UPLC system (Acquity system, Waters, Milford, USA) was coupled to a Xevo TQ-S triple quadrupole mass spectrometer (Waters). Chromatographic separations were carried out using revers phase hybrid column (Kinetex, 2.6 *μ*m, 50 x 2.1 mm; Phenomenex, CA, USA) maintained at 30°C. Amygdalin and paeoniflorin were separated using a gradient elution with a flow rate of 0.5 mL/min. Mobile phase solvent A was 50 mM Ammonium formate (Sigma-Aldrich) in water (pH 8.0) and solvent B was 50 mM Ammonium formate in acetonitrile. The samples were eluted according to the following linear gradient from 5% B buffer to 100% for 10 mins. Ions were generated in positive ionization mode using electrospray ionization interface. Tandem MS analysis was performed using the multi-reaction-monitoring (MRM) mode by monitoring the transition pair of m/z 474.93 → 325.11 for amygdalin and m/z 497.93 → 179.01 for paeoniflorin. The gas flows of desolvation, cone, and nebulizer were set at 650 L/Hr, 150 L/Hr, and 7 bar, respectively.

#### 2.2.2. Cinnamic Acid

Agilent 6410B Triple Quadrupole LC/MS (Agilent Technologies, Wilmington, USA) equipped with an ESI source was employed for the analysis. Cinnamic acid (PubChem CID: 444539, purity 99.0%) was purchased from Sigma-Aldrich and used as reference standard. 1 g of each sample was mixed with 10 mL of methanol and centrifuged. Aliquots of 5 *μ*L of the processed samples were injected into the HPLC system (1200 Series LC, Agilent Technologies, Wilmington, USA) fitted with Phenomenex Kinetex C18 2.6 *μ*m 80 Å 50 x 2.1 mm column, maintained at 35°C. ESI was operating at +3000V and a source temperature of 380°C. Capillary voltage, cone voltage, and source offset were set at 3kV, 30kV, and 30V, respectively. The gas flow of desolvation and the cone was set at 650L/Hr and 150 L/Hr with a nebulizer pressure of 15 bar. A mobile phase composed of 0.1% formic acid in distilled water (Buffer A) and 0.1% formic acid in acetonitrile (Buffer B) was used to separate the analysts and pumped into the ESI chamber at a flow rate of 0.5 mL/min for 20 min. Fragmentor voltage and collision voltage were set at 50V. Detection of the ions was carried out in the multiple-reaction monitoring mode (MRM), by monitoring the transition pairs of m/z 149.1 →131 (cinnamic acid). Data acquisition was performed with the MassHunter Software (Version B.04.00).

### 2.3. Reagents and Cell Culture

LPS, fluoxetine (FXT), and amitriptyline (AMT) were purchased from Sigma-Aldrich. Tin protoporphyrin IX (SnPP; HO-1 inhibitor) and cobalt protoporphyrin IX (CoPP; HO-1 inducer) were purchased from Santa Cruz Biotechnology (San Diego, CA). Murine BV-2 microglial cells were obtained from Dr. SW Chae (Korea Institute of Oriental Medicine) and cultured in Dulbecco's Modified Eagle's medium (DMEM; Lonza, Walkersville, MD, USA) supplemented with 10% fetal bovine serum (FBS; Gibco, Thermo Fisher Scientific, Carlsbad, MA, USA) and 100 *μ*g/mL penicillin-streptomycin (Gibco) at 37°C in a humidified atmosphere containing 5% CO_2_.

### 2.4. Cell Viability Assay

BV2 cells (5 × 10^5^ /mL) were cultured in the presence of GBH (12.5-800 *μ*g/mL) for 24 h and stained with 3-(4,5-dimethylthiazol-2-yl)-5-(3-carboxymethoxyphenyl)-2-(4-sulfophenyl)-2H–tetrazolium (MTS; Promega, USA) for 4 h at 37°C. Absorbance was measured at 490 nm using a microplate reader (Molecular Devices, Sunnyvale, CA, USA).

### 2.5. Nitric Oxide Determination

BV2 cells (2 × 10^5^ /mL) were pretreated with GBH, FXT, or AMT for 1 h and then stimulated with LPS (100 ng/mL) for 24 h. The levels of nitric oxide in the culture supernatant were determined using a nitric oxide (NO) detection kit (iNtRON BioTechnology, Korea), according to the manufacturer's instructions.

### 2.6. ELISA

BV2 cells (2 × 10^5^ /mL) were pretreated with GBH, FXT, or AMT for 1 h and then stimulated with LPS (100 ng/mL) for 16 h. The levels of IL-6, IL-1*β*, TNF-*α*, and IL-10 in the culture supernatant were determined using a commercially available ELISA kit (R&D systems, Minneapolis, MN, USA), according to the manufacturer's protocols.

### 2.7. Real-Time PCR

Total RNA was isolated by using Trizol (Invitrogen) and cDNA synthesis was performed using the PrimeScript™ RT reagent kit (TaKaRa, Shiga, Japan).* IL-1β, IL-6, TNF-α, MIP-1α, MCP-1, IP-10, RANTES, iNOS, COX-2, HO-1*, and* glyceraldehyde-3 phosphate dehydrogenase* (*GAPDH*) mRNA were quantified using a QuantStudio™ 6 Flex real-time polymerase chain reaction (real-time PCR) system (Applied Biosystems, CA, USA) with Power SYBR® Green PCR Master Mix (Applied Biosystems) [22]. Primer sequences are enumerated in Table 1.

### 2.8. Western Blot Analyses

Whole lysates were harvested using a Radioimmunoprecipitation Assay (RIPA) buffer containing protease inhibitor cocktail (Sigma-Aldrich), 1 mM phenylmethylsulfonyl fluoride, and phosphatase inhibitor cocktail set III (Calbiochem, CA, USA). Nuclear extracts were prepared using a nuclear extract kit (Active Motif, Carlsbad, CA, USA.). Equal amounts (20 *μ*g) of protein were separated using 10% sodium dodecyl sulfate-polyacrylamide gel electrophoresis (SDS-PAGE) and transferred to polyvinylidene fluoride (PVDF) membranes (Amersham Biosciences, Piscataway, NJ, USA), which were blocked with 5% skim milk in TBS/T (Tris buffered saline in 0.1% TWEEN® 20) buffer for 1 h. The membranes were then treated overnight with antibodies specific to iNOS, NF-*κ*B p65, HO-1, NRF2, phospho-CREB, CREB, phospho-p38 (Thr180/Tyr182), p38, phospho-Erk (Thr202/Tyr204), Erk, phospho-JNK (Thr183/Tyr185), JNK, phospho-Akt (Ser473), and Akt (Cell Signaling Technology, Beverly, MA). Blots were incubated with horseradish peroxidase- (HRP-) conjugated secondary antibody for 1 h at room temperature. HRP was detected using a chemiluminescent detection reagent (Amersham Biosciences). *β*-actin (Sigma-Aldrich) and proliferating cell nuclear antigen (PCNA; Cell Signaling Technology) were used as loading controls. Chemiluminescence was visualized using an LAS-3000 LuminoImage analyzer (Fujifilm, Tokyo, Japan) [23].

### 2.9. Statistical Analyses

All data are expressed as mean ± standard deviation (SD). One-way analyses of variance (ANOVAs) were performed using GraphPad Prism version 7 (GraphPad Software Inc., San Diego, CA, USA) to assess between-group differences. Multiple group comparisons were performed using one-way ANOVAs, followed by a post-hoc Tukey test.* P* value <0.05 was considered statistically significant.

## 3. Results

### 3.1. LC/MS Analysis of Gyejibokryeong-Hwan

**Figure 1:**
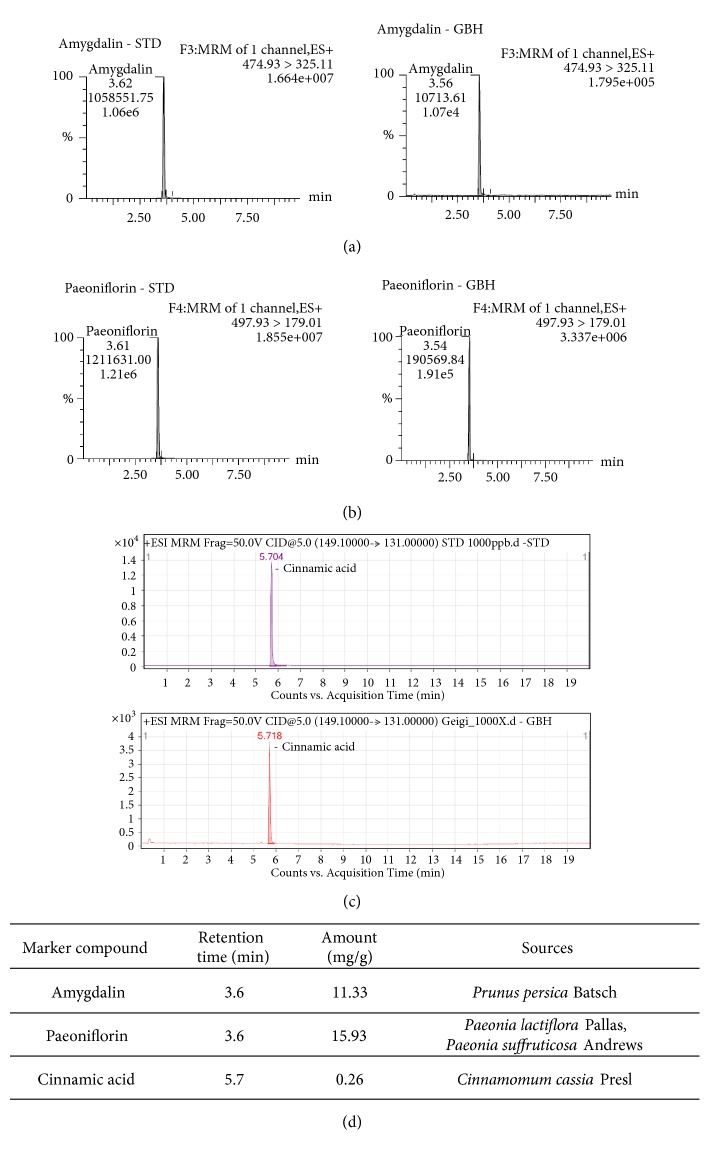
Chromatograms of Gyejibokryeong-hwan (GBH) samples based on liquid chromatography-mass spectrometry (LC/MS) analysis. LC/MS chromatograms of a standard mixture and of the GBH sample revealed (a) amygdalin, (b) paeoniflorin, and (c) cinnamic acid. (d) Identification of GBH components. LC/MS: liquid chromatography-mass spectrometry.

The composition of GBH was verified using LC/MS. This analysis provided chemical information which guaranteed the reproducibility of our experiments in other batches of GBH. Analyses were performed in accordance with the chemical standards described by Kim et al. [13]. Quantitative and qualitative analyses of the three marker compounds (amygdalin, paeoniflorin, and cinnamic acid) in GBH were conducted using the LC/MS method. Each GBH component was identified using MRM mode. The retention times and amounts of each of the three GBH components are shown in Figure 1.

### 3.2. Gyejibokryeong-Hwan Inhibited LPS-Induced NO Production and iNOS and COX-2 Expression in BV2 Microglia

**Figure 2:**
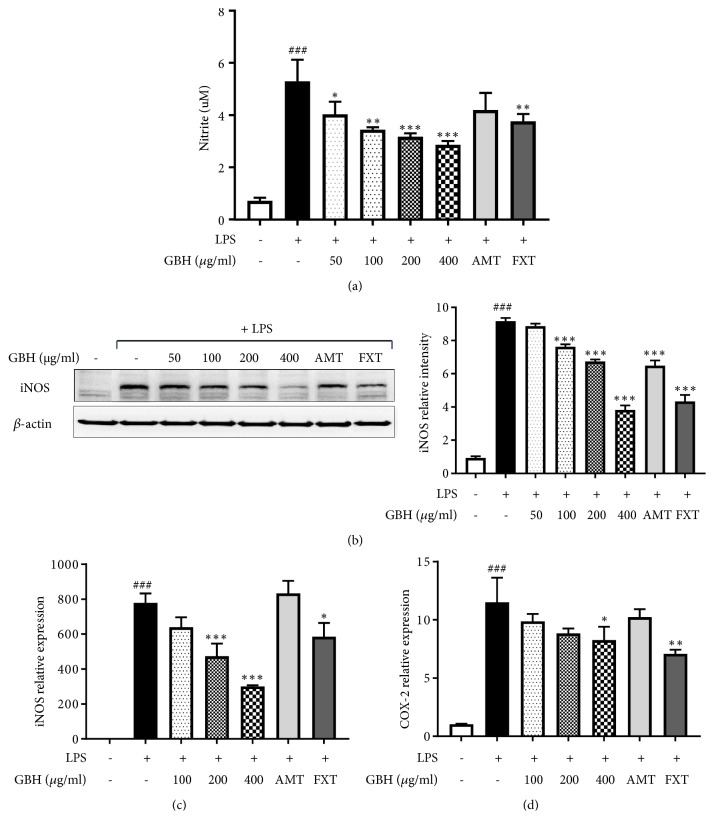
Effect of Gyejibokryeong-hwan on NO production and iNOS and COX-2 expression in BV2 cells. BV2 cells were pretreated with GBH for 1 h and then stimulated with LPS (100 ng/mL) for 24 h. (a) The levels of NO in the cell culture supernatant were measured by NO detection kit. (b) The level of iNOS was determined by western blot analyses. *β*-actin was used as a loading control. The data represent three independent experiments. BV2 cells were pretreated with GBH for 1 h and then stimulated with LPS (100 ng/mL) for 6 h. Levels of (c) iNOS and (d) COX-2 mRNA were determined by real-time PCR. GAPDH was used as a loading control. The data represent the mean ± SD of triplicate determinations (one-way ANOVA;  ^###^p < 0.001 vs. control;  ^*∗*^p < 0.05,  ^*∗∗*^p < 0.01,  ^*∗∗∗*^p < 0.001 vs. LPS-treated control).

We first performed an MTS assay to determine the cytotoxicity and antineuroinflammatory and neuroprotective effects of GBH. The result of this assay demonstrated that GBH does not have cytotoxic effect at up to 800 *μ*g/mL (Table 2). To investigate the effects of GBH on NO production, BV2 cells were treated with LPS (100 ng/mL) in presence or absence of GBH. The production of NO was increased up to seven-fold compared over that of controls, which treatment with GBH (50-400 *μ*g/mL) significantly blocked in a dose-dependent manner (Figure 2(a)). iNOS protein levels were also dramatically increased in LPS-treated controls, and similar to NO, treatment with GBH (400 *μ*g/mL) suppressed these to nearly control levels (Figure 2(b)). Further, as shown in Figures 2(c) and 2(d),* iNOS* and* COX-2* mRNA levels were greatly increased by LPS stimulation. GBH (400 *μ*g/mL) significantly suppressed these* iNOS* and* COX-2* gene expression increases. Furthermore, FXT and AMT (10 *μ*M), positive controls used in this study, also had inhibitory effects on iNOS and NO production. These data suggest that GBH may inhibit NO production through downregulation of* iNOS* and* COX-2*.

BV2 cells were treated with various concentrations of GBH for 24 h, and then cell viability was measured by MTS assay. The data represent the mean ± SD of triplicate determinations. MTS: 3-(4,5-dimethylthiazol-2-yl)-5-(3-carboxymethoxyphenyl)-2-(4-sulfophenyl)-2H–tetrazolium.

### 3.3. Gyejibokryeong-Hwan Inhibited LPS-Induced Proinflammatory Cytokine and Chemokine Expression in BV2 Microglia

**Figure 3:**
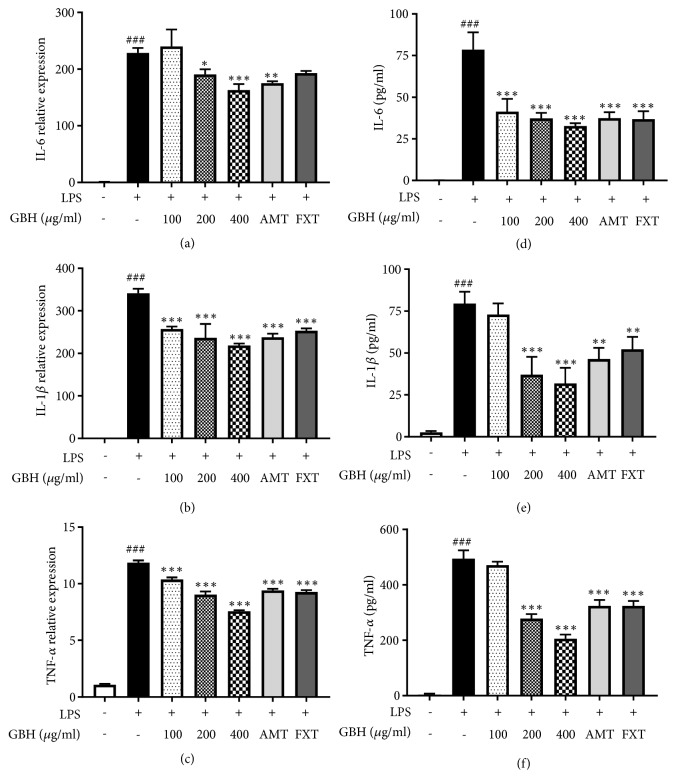
Effect of Gyejibokryeong-hwan on mRNA expression of proinflammatory cytokines in BV2 cells. BV2 cells were pretreated with GBH for 1 h and then stimulated with LPS (100 ng/mL) for 6 h. mRNA expression levels of (a) IL-6, (b) IL-1*β*, and (c) TNF-*α* were determined by real-time PCR. GAPDH was used as a loading control. BV2 cells were pretreated with GBH for 1 h and then stimulated with LPS (100 ng/mL) for 16 h. Levels of (d) IL-6, (e) IL-1*β*, and (f) TNF-*α* in the cell culture supernatant were measured via ELISA. The data represent the mean ± SD of triplicate determinations (one-way ANOVA;  ^###^p < 0.001 vs. control;  ^*∗*^p < 0.05,  ^*∗∗*^p < 0.01,  ^*∗∗∗*^p < 0.001 vs. LPS-treated control).

**Figure 4:**
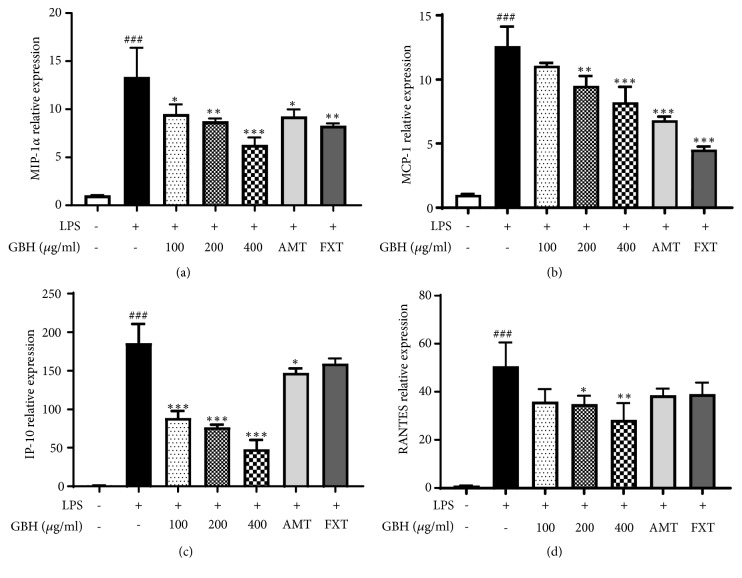
Effect of Gyejibokryeong-hwan on mRNA expression of proinflammatory chemokines in BV2 cells. BV2 cells were pretreated with GBH for 1 h and then stimulated with LPS (100 ng/mL) for 6 h. Levels of (a) MIP-1*α*, (b) MCP-1, (c) IP-10, and (d) RANTES mRNA were determined by real-time PCR. GAPDH was used as a loading control. The data represent the mean ± SD of triplicate determinations (one-way ANOVA;  ^###^p < 0.001 vs. control;  ^*∗*^p < 0.05,  ^*∗∗*^p < 0.01,  ^*∗∗∗*^p < 0.001 vs. LPS-treated control).

The LPS-stimulated microglia produce high levels of cytokines such as IL-6, IL-1*β*, and TNF-*α*. The mRNA levels of* IL-6*,* IL-1β*, and* TNF-a*, which were dramatically increased by LPS stimulation, were significantly decreased by GBH in a dose-dependent manner (Figures 3(a)–3(c)). FXT and AMT (10 *μ*M) also showed inhibitory effect on mRNA levels of* IL-6*,* IL-1β*, and* TNF-α*. Furthermore, production of IL-6, IL-1*β*, and TNF-*α* was markedly increased in LPS-treated control; however, the pretreatment with GBH significantly inhibited the cytokine levels in a dose-dependent manner (Figures 3(d)–3(f)). The mRNA levels of chemokines such as* MIP-1α*,* MCP-1*,* IP-10*, and* RANTES* were greatly increased in LPS-treated control, but the presence of GBH significantly suppressed the levels in a dose-dependent manner (Figures 4(a)–4(d)). These data suggest that GBH might downregulate LPS-induced proinflammatory cytokines and chemokines.

### 3.4. Gyejibokryeong-Hwan Inhibited LPS-Induced Activation of MAPK, PI3K/Akt, and NF-*κ*B Inflammatory Pathways in BV2 Microglia

**Figure 5:**
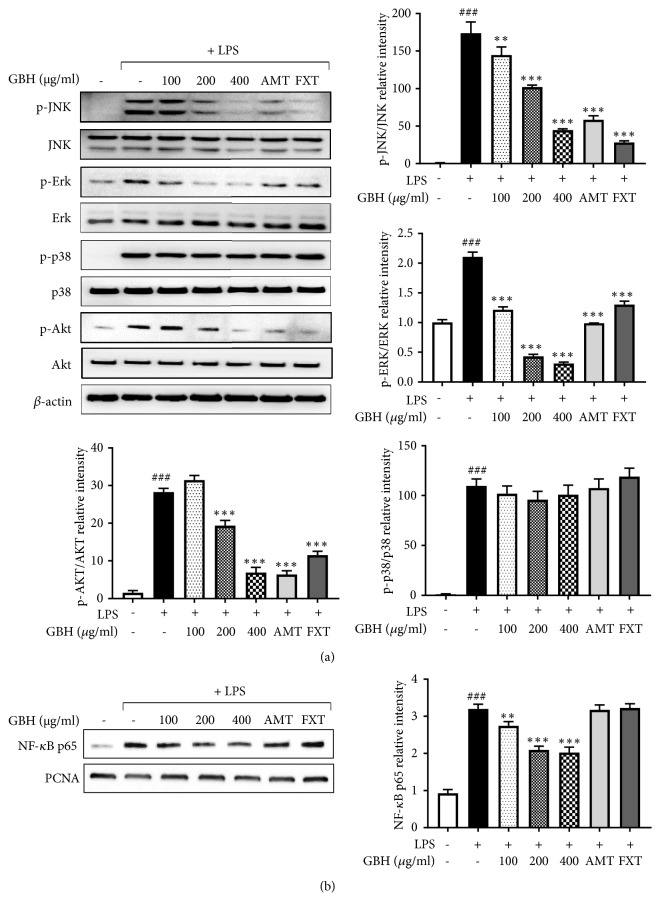
Effect of Gyejibokryeong-hwan on the phosphorylation of MAPK and Akt and NF-*κ*B activity in BV2 cells. (a) BV2 cells were pretreated with GBH for 1 h and then stimulated with LPS (100 ng/mL) for 15 min. Levels of p-JNK, JNK, p-Erk, Erk, p-p38, p38, p-Akt, and Akt were determined by western blot analyses. *β*-actin was used as a loading control. (b) BV2 cells were pretreated with GBH for 1 h and then stimulated with LPS (100 ng/mL) for 1 h. Nuclear extracts were analyzed by western blot analysis using an NF-*κ*B p65 antibody. PCNA was used as a loading control. The data represent three independent experiments. The data are expressed as the mean ± SD of triplicate determinations (one-way ANOVA;  ^###^p < 0.001* vs*. control;  ^*∗∗*^p < 0.01,  ^*∗∗∗*^p < 0.001* vs*. LPS-treated control).

MAPKs, PI3K/Akt, and NF-*κ*B play important roles in the signaling pathways that induce a neuroinflammatory response in microglia [24]. Given this, the effects of GBH on MAPKs, PI3K/Akt, and NF-*κ*B pathways were examined here. As shown in Figure 5(a), the levels of phosphorylated JNK in control cells were minimal, while treatment with LPS dramatically increased these levels. Additionally, GBH lowered phosphorylated JNK levels, while total JNK levels were unchanged. Levels of phosphorylated Erk were dramatically increased after LPS, increases corrected by GBH treatment. However, levels of phosphorylated p-38 MAPK were unchanged by GBH. Furthermore, the phosphorylation of Akt was significantly increased by LPS treatment, while total Akt was not affected. With GBH, levels of phosphorylated Akt were significantly reduced. NF-*κ*B is a key transcription factor that modulates iNOS and proinflammatory cytokine and chemokine gene expression in microglia. Western blot analyses using nuclear extract from treated and control BV2 cells indicated that expression of p65, a component of NF-*κ*B, significantly increased by LPS stimulation. Pretreatment with GBH significantly inhibited expression of these factors in the nucleus (Figure 5(b)). These data suggest that GBH may interfere with Erk, JNK, and Akt to facilitate altered NF-*κ*B pathway signaling and inhibit neuronal proinflammatory responses to LPS stimulation.

### 3.5. Gyejibokryeong-Hwan Increased IL-10 and HO-1 Expression via Upregulation of NRF2/CREB Pathways in BV2 Microglia

**Figure 6:**
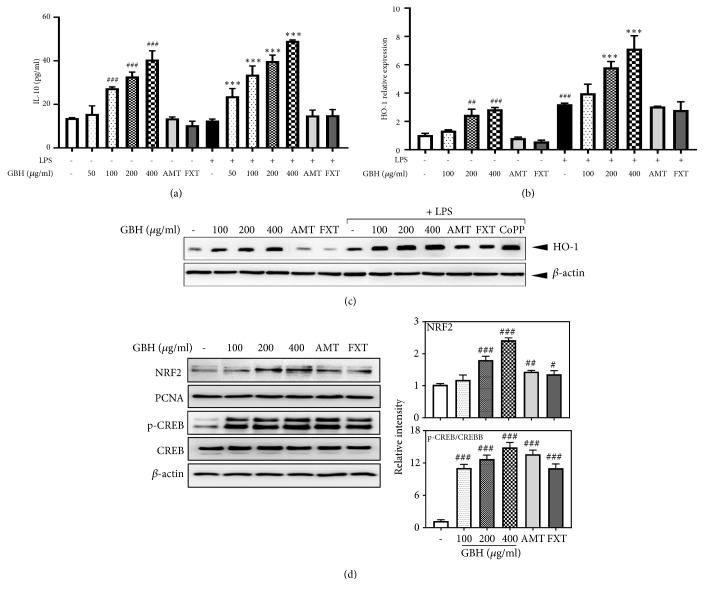
Effect of Gyejibokryeong-hwan on IL-10 production and HO-1 expression in BV2 cells. (a) BV2 cells were pretreated with GBH for 1 h and then stimulated with LPS (100 ng/mL) for 16 h. Levels of IL-10 in the cell culture supernatant were measured via ELISA. (b) BV2 cells were pretreated with GBH for 1 h and then stimulated with LPS (100 ng/mL) for 6 h.* HO-1* mRNA levels were determined by real-time PCR.* GAPDH* was used as a loading control. (c) BV2 cells were pretreated with GBH for 1 h and then stimulated with LPS (100 ng/mL) for 12 h. The level of HO-1 was determined by western blot. *β*-actin was used as a loading control. (d) BV2 cells were pretreated with GBH for 6 h. Nuclear extracts were analyzed by western blot using an NRF2 antibody. PCNA was used as a loading control. BV2 cells were pretreated with GBH for 30 min. Levels of p-CREB and CREB were determined by western blot analyses. *β*-actin was used as a loading control. The data represent three independent experiments. The data are expressed as the mean ± SD of triplicate determinations (one-way ANOVA;  ^#^p < 0.05,  ^##^p < 0.01,  ^###^p < 0.001 vs. control;  ^*∗∗∗*^p < 0.001 vs. LPS-treated control).

IL-10 and HO-1 act as anti-inflammatory and antioxidant modulators via their upregulation of NRF2/CREB pathways in microglia and other cells [25]. Given this, we examined the effect of GBH on IL-10 and HO-1 expression in LPS-treated and control BV2 cells. Levels of IL-10 in controls (13.7 pg/mL) increased in a dose-dependent fashion with GBH. LPS-treated cells produced similar levels of IL-10 as controls, though cells treated with both LPS and GBH had increased IL-10 levels compared to those treated with GBH only (Figure 6(a)). Next, we examined mRNA and protein expression of HO-1, both of which were significantly increased in LPS-treated control cells over true control. GBH and LPS treatment increased HO-1 expression over levels in GBH control cells (Figures 6(b) and 6(c)). CoPP (20 *μ*M) was used as a western blot positive control to induce HO-1 expression (Figure 6(c)). Furthermore, GBH increased nuclear translocation of NRF2 and phosphorylated CREB, which acts as an upstream modulator of HO-1 expression (Figure 6(d)). These data suggest that GBH might upregulate HO-1 via NRF2/CREB pathways involved in the neuroprotective effects in BV2 microglia.

### 3.6. HO-1 Mediates the Effects of Gyejibokryeong-Hwan on NO Production and Proinflammatory Cytokine mRNA in BV2 Microglia

**Figure 7:**
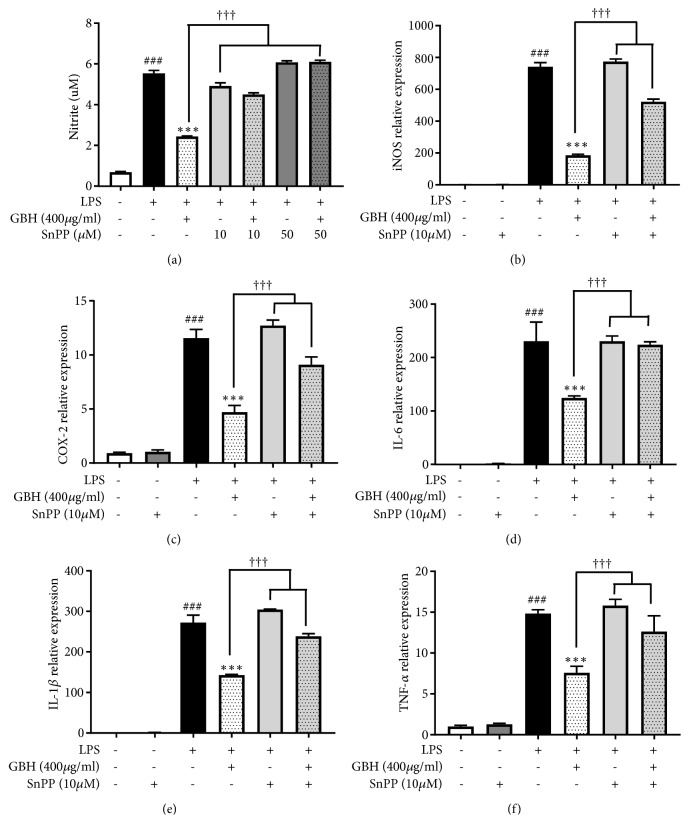
HO-1 mediates the effect of Gyejibokryeong-hwan on NO production and mRNA expression of proinflammatory cytokines in BV2 cells. (a) BV2 cells were pretreated with GBH for 1 h in the presence or absence of SnPP (10 *μ*M) and then stimulated with LPS (100 ng/mL) for 24 h. Levels of NO in the cell culture supernatant were measured by an NO detection kit. BV2 cells were pretreated with GBH for 1 h in the presence or absence of SnPP (10 *μ*M) and then stimulated with LPS (100 ng/mL) for 6 h. Levels of (b)* iNOS*, (c)* COX-2*, (d)* IL-6, *(e)* IL-1β,* and (f)* TNF-α* mRNA were determined by real-time PCR.* GAPDH* was used as a loading control. The data represent the mean ± SD of triplicate determinations (one-way ANOVA;  ^###^p < 0.001 vs. control;  ^*∗∗∗*^p < 0.001 vs. LPS-treated control; ^†††^p < 0.001 vs. LPS+GBH).

To confirm the effects of GBH on proinflammatory signaling pathways, we examined whether HO-1 mediates the effect of GBH on NO production and proinflammatory cytokines gene expression. This was assessed by cotreating cells with SnPP, an inhibitor of HO-1 activity. We found that GBH significantly reduced NO production and* iNOS*,* COX-2*, and proinflammatory cytokine gene expression levels. However, SnPP did not reverse the inhibitory effects of GBH on NO production and* iNOS*,* COX-2*, and proinflammatory cytokine mRNA levels (Figures 7(a)–7(f)). Collectively, these results suggest that GBH act as an antineuroinflammatory and neuroprotective agent in BV2 microglia via upregulation of HO-1.

## 4. Discussion

In adults, microglia represent about 10% of the cells in the brain and spinal cord [26]. Microglia are involved in innate immunity and regulate cytokines and inflammatory processes in the brain [27]. When activated by infection or tissue damage, microglia produce inflammatory factors, including proinflammatory cytokines, chemokines, and reactive oxidant species, which can cause neuronal toxicity and degeneration [28]. Neuroprotective factors such as the anti-inflammatory cytokine IL-10 and HO-1 inhibit neuroinflammation and neuronal cell death [29].

Moreover, in recent studies, microglia have been suggested as an important factor leading to depression by regulating neuroimmune pathways affecting neuroplasticity [1, 2]. Various studies have been conducted to demonstrate this antidepressant effect using BV2 microglia cells [30, 31]. In a previous study by our group, GBH was shown to inhibit neuroinflammation in the hippocampus of a mouse model of reserpine-induced depression [19]. Thus, the present* in vitro *study sought to understand the mechanisms of the antineuroinflammation effects of GBH observed in our previous study on* in vivo* models of depression.

**Figure 8:**
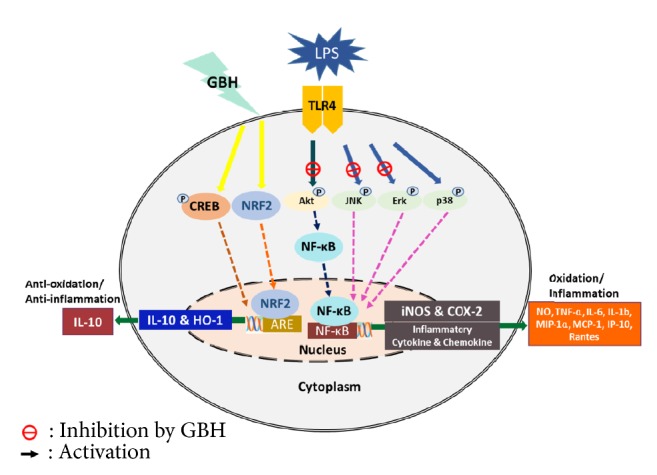
The proposed mechanism underlying the antineuroinflammatory and neuroprotective effects of Gyejibokryeong-hwan in lipopolysaccharide-stimulated BV2 microglia.

Specifically, antidepressants are known to inhibit the production of proinflammatory cytokines and suppress microglial activation [32–35]. FXT (selective serotonin reuptake inhibitors, SSRI) and AMT (tricyclic antidepressants, TCA) have been reported to induce anti-inflammatory effects in activated microglia and were, therefore, used as positive controls in the present study [36–38]. In the experiments presented here, we demonstrated a molecular mechanism for the antineuroinflammatory and neuroprotective effects of GBH in LPS-activated microglia. The proposed mechanism underlying the effects of GBH is summarized in Figure 8.

NO is a free radical that plays an important role in various physiological and pathological processes. Under physiological conditions, NO is involved in defense against tumors, parasites, and bacteria [39]. However, when it is overproduced, NO acts as a toxic radical, causing damage to cells and tissues [40]. The expression of iNOS and COX-2, key enzymes in NO production, has been reported to be increased in activated glial cells [41]. In the present study, we found that GBH inhibited NO production by suppressing iNOS and COX-2 mRNA and protein levels.

Proinflammatory cytokines play an important role in the immune response, and their excessive production has been reported to exacerbate various inflammatory responses such as neurodegenerative disorders, multiple sclerosis [42]. Based on the results reported here, mRNA expression levels of TNF-*α*, IL-1*β*, and IL-6 were inhibited by GBH. Furthermore, chemokines increase the infiltration of inflammatory immune cells in the brain [43]. Such recruitment may contribute to neuropathologies, including neuropathic pain after nerve injury [44]. Our data clearly demonstrated that BV2 cell treatment with GBH significantly reduced mRNA levels of IP-10, MIP-1*α*, MCP-1, and RANTES in LPS-stimulated microglia.

Activation of Toll-like receptors (TLR) 4/MyD88 by LPS stimulation of microglia triggers NF-*κ*B, MAPKs, and PI3K/Akt. With phosphorylation of IkB, an NF-*κ*B inhibitory protein that masks the nuclear localization signal of NF-*κ*B, the protein is ubiquitinated and degraded, freeing it to translocate into the nucleus and initiate the transcription of inflammatory genes [45–47]. The present study demonstrated that GBH suppressed the activation of NF-*κ*B and the phosphorylation of JNK and Erk in specific MAPKs and Akt in LPS-stimulated BV2 cells. Taken together, those anti-inflammatory effects of GBH might lead to diminished levels of neuropathological factors such as NO, proinflammatory cytokines, and chemokines, as also evidence here.

Induction of HO-1 can serve as a potent protector against inflammation and cell death via the generation of its by-products, including carbon monoxide, biliverdin, and iron [48]. Recent studies have shown that NRF2 and CREB are important upstream contributors to HO-1 induction [25]. In particular, NRF2 has been reported to inhibit NF-*κ*B activation [49]. In the present study, we demonstrated that GBH significantly increased HO-1 expression via increased nuclear translocation of NRF2 and CREB phosphorylation. Furthermore, levels of IL-10, a canonical anti-inflammatory cytokine [50], were increased by GBH in both the presence and absence of LPS. Indeed, the use of an HO-1 antagonist also reversed the effect of GBH on NO production, as well as on increased iNOS, COX-2, and proinflammatory cytokine mRNA levels. The regulation and balance of NF-*κ*B and NRF2 levels is critical to neuroinflammatory processes and to neuroprotection [49]. Despite this understanding and the molecular markers implicated in* in vitro* work here, further studies are needed to fully elucidate the coregulation, negative feedback, and competitive binding mechanisms by which these markers behave* in vivo.*

## 5. Conclusions

Recently, scientific work has begun to focus on the anti-inflammatory and antioxidant properties of natural medicines, which may be effective in treating major neurodegenerative, autoimmune, and cancerous disease states with a minimal risk of side effects [51]. GBH, one such active, natural compound, has been reported to be safe at a dose of 500 mg/kg/day, regardless of sex [52, 53]. Before the use of this compound can be fully realized in a clinical context, however, studies must address its molecular mechanisms and activity, as we have endeavored to do here. Taken together, our results suggest that GBH may act as a neuroprotective agent by downregulating neuroinflammation in activated, cultured microglia. GBH may thus have significant therapeutic potential for the treatment of depression associated with microglial activation status without serious side effects.

## Figures and Tables

**Table 1:** The sequences of the real-time PCR primers.

Gene	Sequence
mouse *IL-1β*	forward, 5′- GCTGAAAGCTCTCCACCTCA -3′
	reverse, 5′- AGGCCACAGGTATTTTGTCG -3′

mouse *IL-6*	forward, 5′- GAGGATACCACTCCCAACAGACC -3′
	reverse, 5′- AAGTGCATCATCGTTGTTCATACA -3′

mouse *TNF-α*	forward, 5′- AGACCCTCACACTCAGATCATCTTC -3′
	reverse, 5′- CCACTTGGTGGTTTGCTACGA -3′

mouse *HO-1*	forward, 5′- AGCCCCACCAAGTTCAAACA -3′
	reverse, 5′- CATCACCTGCAGCTCCTCAA -3′

mouse *MIP-1a*	forward, 5′- CCCAGCCAGGTGTCATTTTCC -3′
	reverse, 5′- GCATTCAGTTCCAGGTCAGTG -3′

mouse *MCP-1*	forward, 5′- GCTCAGCCAGATGCAGTTAA -3′
	reverse, 5′- TCTTGAGCTTGGTGACAAAAACT -3′

mouse *IP-10*	forward, 5′- GAATCCGGAATCTAAGACCATCAA -3′
	reverse, 5′- GTGCGTGGCTTCACTCCAGT -3′

mouse *RANTES*	forward, 5′- CAGCAGCAAGTGCTCCAATCTT -3′
	reverse, 5′- TTCTTGAACCCACTTCTTCTCTGG -3′

mouse *iNOS*	forward, 5′- GAATCTTGGAGCGAGTTGTGGA -3′
	reverse, 5′- GTGAGGGCTTGGCTGAGTGAG -3′

mouse *COX-2*	forward, 5′- TGGGGTGATGAGCAACTATT-3
	reverse, 5′- 5-AAGGAGCTCTGGGTCAAACT-3

mouse *GAPDH*	forward, 5′- AAGGTGGTGAAGCAGGCAT -3′
	reverse, 5′- GGTCCAGGGTTTCTTACTCCT -3′

**Table 2:** Effects of Gyejibokryeong-hwan on cell viability.

Concentration (*μ*g/mL)	Cell viability (%)
	GBH
0	101.15 ± 1.03
12.5	107.35 ± 0.68
25	107.82 ± 1.02
50	109.50 ± 1.45
100	110.05 ± 0.93
200	106.01 ± 1.83
400	107.65 ± 3.43
800	107.37 ± 2.96

## Data Availability

The datasets used and/or analyzed in the current study are available from the corresponding author upon reasonable request. The role of the funding body in the design of the study and collection, analysis, and interpretation of data and in writing the manuscript should be declared in this request.
